# Exploring the mediating roles of motivation and boredom in basic psychological needs and behavioural engagement in English learning: a self-determination theory perspective

**DOI:** 10.1186/s40359-025-02524-3

**Published:** 2025-02-28

**Authors:** Honggang Liu, Ying Wang, Haoyue Wang

**Affiliations:** 1 School of Foreign Languages, Soochow University, Suzhou, 215006 China; 2 Foreign Languages College, Inner Mongolia University, Hohhot, Inner Mongolia Autonomous Region, 010021, China; 3 School of Foreign Languages, Boda College of Jilin Normal University, Siping, Jilin Province 136000 China

**Keywords:** Basic psychological needs, Self-determined motivation, Boredom, Behavioural engagement, Mediation analysis

## Abstract

In second language acquisition research, the psychological factors associated with language learning have been a prominent focus. The affective turn in and introduction of positive psychology in this field have further boosted research on the roles of positive learner psychological factors (e.g. motivation) in performance (e.g. engagement). However, the theoretical lens for investigating these variables requires further clarification, and the roles of some variables (e.g. boredom) in language learning have been under-researched. Guided by this background, this study aims to explore the complex relationships between learners’ basic psychological needs, self-determined motivation, boredom and behavioural engagement among 687 Chinese senior high school EFL learners from the perspective of self-determination theory (SDT). Quantitative data collection and analysis revealed that students’ basic psychological needs directly predicted behavioural engagement. Basic psychological needs also indirectly predicted behavioural engagement through the simple mediation of boredom and the chain mediation of self-determined motivation and boredom. However, the simple mediation of self-determined motivation in the relationship between basic psychological needs and behavioural engagement was non-significant. The findings enrich the application of SDT in the language learning engagement research, providing valuable implications for teachers and educators.

**Supplementary Information:**

The online version contains supplementary material available at 10.1186/s40359-025-02524-3.

## Introduction

Among researchers studying second language acquisition (SLA), language learner psychological factors have been a critical topic [1, 2]. With the emergence of the affective turn [3] and the introduction of positive psychology [4], studies on language learner psychological factors, especially positive ones, such as positive emotions (e.g [5, 6])., enjoyment (e.g [7, 8])., motivation (e.g [9–12])., resilience (e.g [13–15]). and buoyancy (e.g [16–18])., have flourished over the last decade. As a critical variable relating to language learning performance [19], learning engagement has attracted considerable attention from SLA researchers. For instance, the factors influencing learning engagement have been extensively researched, exemplified by motivation (e.g [20, 21]). and grit (e.g [22, 23]). In addition, following the holistic view of foreign language emotion research [4], the impact of negative emotions, such as anxiety [16, 18, 24], burnout [25] and boredom [9, 26, 27], on language learning engagement has been a salient focus in SLA (e.g [28, 29]). As learning engagement is a multidimensional construct with behavioural engagement as its core component [30–32], studies have also examined the factors influencing behavioural engagement in SLA (e.g [33]).

However, some studies on language learning engagement have not had a clear theoretical basis. The research focus, based on the empirical findings of previous studies, has been on describing language learning engagement levels and their relationships with other contextual and psychological factors without the guidance of related theories. The absence of a solid theoretical foundation would be detrimental not only to the systematic summary of related studies [34] but also to the proliferation of foreign language learning engagement research. In response, self-determination theory (SDT) concerns how people’s behaviours and achievements are influenced by their psychology and environment, offering a solid theoretical foundation for exploring the association between engagement and other factors in foreign language learning [35]. Applying SDT, a significant research result in the general education and foreign language learning field is that learners’ basic psychological needs, including autonomy, competence and relatedness, are essential to explaining their learning behaviours, such as engagement (e.g [36, 37]).

Nevertheless, to our knowledge, the complex relationship between the two factors remains under-researched. For example, studies have investigated the relationship between learners’ basic psychological needs, motivation and engagement in the field of general education (e.g [38–40])., but the English as a foreign language (EFL) learning context has received less attention. Likewise, there is limited research on the relationships between basic psychological needs, engagement and emotions. Only enjoyment and anxiety have attracted EFL researchers’ attention regarding the above connection (e.g [41]). Therefore, it appears that motivation, emotions and basic psychological needs and engagement among EFL learners need further investigation.

In the Chinese senior high school EFL context, English is a compulsory subject and a major part of the College Entrance Examination, which creates stress and challenges for many students. According to SDT, such stress and challenges are likely to inhibit students’ free choice in EFL learning, the feeling of being competent and the relaxed learning atmosphere, among others. In this high-stakes context, how students’ basic psychological needs are satisfied and their effects on learning psychology (e.g. motivation and boredom) and behaviours (e.g. behavioural engagement) require further investigation.

In light of the above research background and SDT principles, this study aims to explore the complex mechanisms between basic psychological needs, motivation, boredom and behavioural engagement among Chinese senior high school EFL learners to extend the theoretical background of foreign language learning research and boost the research on factors influencing EFL engagement.

## Literature review

### Self-determination theory

SDT [42, 43] is a theory of human motivation proposed by American psychologists Edward Deci and Richard Ryan in the 1980s and later developed into a macro-theory of human motivation and well-being through improvement, covering six mini-theories: cognitive evaluation theory, organismic integration theory, basic psychological needs theory, causality orientations theory, goal contents theory and relationships motivation theory [43]. It elaborates on the types of human behavioural motivation as well as the generation of human behavioural motivation and its influence on human psychology, behaviours and well-being [44].

It has become necessary for people to learn a foreign language, and the motivation to do so varies among individuals. Noels et al. [45] found that SDT covers various motivations (e.g. making more friends and making better achievements) and their production and influence on the learning process and outcomes, and they accordingly introduced SDT in the foreign language learning field. Consequently, studies on foreign language learning motivation have been advanced from the lens of SDT (e.g [9, 46, 47]). Thus, it has become a comprehensive research framework for exploring foreign language learning motivation and its antecedents and outcomes [34, 48].

The current study focuses on the antecedent (i.e. basic psychological needs) and outcomes (i.e. boredom and behavioural engagement) of EFL motivation in a Chinese senior high school context. According to SDT, when an individual’s basic psychological needs are satisfied, their motivation to perform an activity becomes self-determined, thereby generating positive psychology and positive behaviours [42, 43]. Therefore, SDT justifies the choice to examine the variables under investigation in this study. Specifically, the two mini-theories of basic psychological needs theory and organismic integration theory are integrated in the present study to explore the complex relationships between basic psychological needs, motivation, boredom and behavioural engagement. As applying SDT entails “articulating a clear rationale for including the concepts or constructs of multiple mini-theories within the design of a single study” [35, p. 25], the basic psychological needs theory will be explained first below.

### Basic psychological needs

Basic psychological needs theory claims that satisfying basic psychological needs is associated with individuals’ well-being [43]. This includes the needs for autonomy, competence and relatedness. Autonomy refers to the psychological state of the willingness to act. Under the satisfaction of autonomy, language learners will voluntarily engage in foreign language learning activities. Competence refers to the feeling of control over one’s actions. Language learners will be aware that they can accomplish tasks related to foreign language learning and recognize their improvement in foreign language acquisition when their need for competence is satisfied. Relatedness comprises the senses of belonging and connectedness, and learners whose need for relatedness is satisfied will perceive others’ care and support in their foreign language learning. The above three basic psychological needs are interrelated, universal and situation-specific. They are prevalent in different cultures, but they will vary in different periods and situations. The basic psychological needs theory also claims that satisfying the three basic psychological needs is optimal for individual development. That is, individuals who are satisfied with their autonomy, competence and relatedness will achieve self-development, experience positive affect and actively engage in activities around them. Based on the above principles, scholars have investigated the relationships between foreign language learners’ basic psychological needs and other psychological and behavioural factors.

### Motivation

Motivation is “a cumulative arousal, or want, that we are aware of” [49, p. 209]. The organismic integration theory in SDT classifies human motivation into three types: amotivation, intrinsic motivation and extrinsic motivation. Amotivation refers to actions that lack intention. Intrinsic motivation refers to actions performed due to interest and enjoyment. Extrinsic motivation refers to actions motivated by external reasons rather than internal joy and satisfaction. Regarding the degrees of internalization and integration of external regulations as part of the self, the organismic integration theory further classifies extrinsic motivation into four subtypes: external regulation, introjected regulation, identified regulation and integrated regulation (ranked from least to most self-determination). If individuals are externally regulated, external stimuli compel them to act. Introjected individuals avoid experiencing negative psychological states. Individuals with identified regulation perform actions when they recognize their value and significance. People with integrated regulation undertake activities because they consider those activities to be part of themselves – something generally neglected in educational contexts, especially among lower-grade students, due to its indistinctive feature ( [50] cited from [45]). Therefore, integrated regulation was not involved in this study. Meanwhile, the current study did not include amotivation because the participants in this study differ in the degree of self-determined motivation rather than being motivated or not. Intrinsic regulation and identified regulation are both highly self-determined and are named autonomous motivation. Introjected and externally regulated motivations are less self-determined and are called controlled motivation.

According to SDT, basic psychological needs directly influence motivation, which has been confirmed in the domain of foreign language learning. Noels [51] investigated 322 students learning Spanish as a second language and found that the needs for autonomy and competence predicted intrinsic motivation; autonomy also strongly predicted identified regulation. The predictive power of the need for competence for intrinsic motivation was more robust than that for extrinsic motivation. Carreira [52] obtained similar results among 505 Japanese primary school EFL learners; they identified stronger correlations between basic psychological needs and highly self-determined motivation. Tanaka [53] confirmed Noels’ [51] finding regarding the predictive power of the needs for autonomy and competence for autonomous motivation in a demotivating vocabulary learning context among 155 Japanese non-English major EFL learners. The study also revealed that negative peer influence negatively predicted autonomous motivation, whereas positive peer influence predicted introjected motivation. The close link between basic psychological needs and highly self-determined motivation was corroborated by Joe et al. [54] in the Korean secondary EFL context and by Alamer [55] among English majors in the Saudi Arabian context.

### Boredom

Emotions (e.g. anxiety) are essential in learning a foreign language [56, 57]. Previous studies have shown the direct predictability of basic psychological needs and self-determined motivation for anxiety. Noels et al. [45] verified the negative relation between highly self-determined motivation and anxiety. Alamer and Almulhim [58] presented a positive correlation between controlled motivation and anxiety and a negative correlation between autonomous motivation and anxiety. Nonetheless, controlled motivation significantly predicted general anxiety. Additionally, the need for competence predicted psychological, achievement and general anxieties. The need for relatedness predicted psychological and social anxieties. Khodadady and Khajavy [59] obtained similar results among 264 Iranian EFL learners.

Moreover, Alamer and Lee’s [60] longitudinal study uncovered the moderating role of self-determined motivation in the relationship between EFL anxiety and achievement among 226 Saudi Arabian university learners. Likewise, Wang and Liu [9] confirmed the mediating role of boredom in the relationship between autonomous learning motivation and engagement in the Chinese senior high school EFL context. These two studies indirectly verified the relationship between self-determined motivation and emotions. To summarize, although the effects of basic psychological needs and self-determined motivation on EFL anxiety have been explored, there is limited research on the influence of basic psychological needs and self-determined motivation on other emotions, such as boredom.

Boredom is a negative emotion defined as “the aversive experience of having an unfulfilled desire to be engaged in satisfying activity” [61, p. 69], including disengagement, high arousal, low arousal, low time perception and inattention [61]. In educational psychology, boredom directly arises from the experience of learning and classroom-related activities [62]. It features multiple components, encompassing affective, cognitive, physiological and motivational elements [63]. When learners experience boredom, they have unpleasant feelings, low time perception or lack of mental stimulation, exhaustion and disengagement from learning activities. Based on the above elaboration, in the current study, boredom is a negative emotion generated from the unfulfilled desire to engage in satisfying EFL learning activities. It also includes affective, cognitive, physiological and motivational components.

With the accelerating emotional turn [64] and positive turn [4] in applied linguistics, EFL scholars have begun to uncover the positive roles of positive psychological factors in the EFL process. However, they have paid limited attention to negative emotions other than anxiety, the long-term focus of researchers in both the general education and EFL fields. Although some negative emotions have gradually attracted EFL researchers’ attention in recent years, such as guilt and shame [65] and boredom [66, 67], they need further investigation. Most studies have assessed university students and have lacked a theoretical perspective. Some of these studies revealed, for instance, that high task challenges, monotonous learning activities [68] and peer involvement [69] were responsible for boredom in the language classroom, which could also be explained from the perspective of basic psychological needs theory, which is closely related to learners’ inclination towards multiple choice, moderately challenging tasks and an active learning atmosphere. Considering that boredom is prevalent and silent in the EFL process, thus easily leading to negative learning psychology [70], it is necessary to closely examine its antecedents and outcomes.

### Behavioural engagement

Learning engagement is a multidimensional construct comprising behavioural, cognitive, affective and social or agentive engagement [27, 71, 72]. Behavioural engagement, as one of the core components of learning engagement, externally reflects the state of engagement, characterized by learners’ effort, attention and participation in the learning process [72–77]. It is the carrier of other dimensions of learning engagement [30, 31] and is the only observable and the most distinguishable dimension among learner psychological factors (e.g. motivation) [32]. Additionally, some studies (e.g [28]). have clarified that among the multiple dimensions of engagement, behavioural engagement has the most predictive power concerning language learning achievement. Therefore, this study focuses on behavioural engagement to enquire into its antecedents.

Although EFL behavioural engagement has recently received scant attention, many studies have investigated the relationship between basic psychological needs, self-determined motivation, emotions and the indicators of behavioural or general learning engagement. In general education, Reeve [36] determined that students’ basic psychological needs were direct antecedents of learning engagement. Evidence of this has also emerged in the field of foreign language learning. Alamer’s [55] investigation revealed the direct relationship between basic psychological needs and effort in vocabulary learning in the Saudi Arabian EFL learning context. Zhou et al. [37] corroborated the above link among 686 Chinese university EFL learners.

As learning engagement is the external manifestation of learning motivation [36, 78], scholars have explored the relationship between self-determined motivation and behavioural engagement. Chen and Kraklow’s [79] survey revealed that intrinsic motivation and external regulation positively influenced effort among 276 Taiwanese university EFL learners. Oga-Baldwin and Fryer [80] adopted a person-centred approach and uncovered that 513 students’ highly self-determined motivation promoted overall engagement over time. Noels et al.’s [81] longitudinal study also demonstrated the positive relation between self-determined motivation and effort in learning French as a second language. Wang and Liu’s [9] cross-sectional survey affirmed the above result. However, studies have also noted the predictive power of controlled motivation. For instance, Sanjaya et al. [82] disclosed the solid predictive value of controlled motivation over autonomous motivation in the Indonesian EFL context.

Studies have also substantiated the impact of emotions on EFL behavioural engagement [18]. However, explorations of the relationship between boredom and EFL behavioural engagement are limited. Feng and Hong’s [33] investigation of 633 Chinese senior high school EFL learners showed a significant and negative correlation between anxiety and behavioural engagement. Liu, Li and Fang’s [27] investigation of 1,157 Chinese secondary EFL learners verified the negative correlation between boredom and behavioural engagement. Another study by Hamedi et al. [28] yielded similar results in EFL reading. The study also presented the more substantial predictive value of boredom, compared to enjoyment and anxiety, on engagement.

### Hypothesized model of the study

To summarize the above studies, behavioural engagement is the core component of EFL learning engagement, reflected by effort, attention and participation in English learning. It is affected by various psychological factors, such as learners’ basic psychological needs, motivation and emotions. However, previous studies also highlighted the scant attention paid to EFL behavioural engagement from the perspective of SDT, although they investigated the relationships between learner psychological factors and EFL engagement or the indicators of behavioural engagement by applying some principles of SDT. However, SDT is a macro-theory that provides a comprehensive research framework for examining the language learning process [34, 48] and offers valid theoretical guidance for a profound examination of the complex language learning process. Therefore, it is necessary to examine the explanatory power of SDT further.

The associations between variables in this study are assumed on the basis of SDT claims and empirical evidence. Firstly, the basic psychological needs theory claims that individuals’ satisfaction of basic psychological needs is closely related to the development of well-being and absence of ill-being [43], which covers positive psychological traits (e.g. self-determined motivation and positive emotions) and behaviours (e.g. engagement) [83, 84]. Guided by the principle of basic psychological needs theory, the present study operates under the assumption that senior high school EFL learners with satisfied basic psychological needs will produce more self-determined motivation, less boredom and more engagement. Furthermore, according to the self-determination continuum of motivation elaborated in organismic integration theory, the degree of self-determination of motivation is negatively related to negative emotions and positively connected to active activity engagement [43, 85]. On these grounds, the authors postulate that self-determined motivation will negatively predict boredom and engagement.

**Fig. 1:**
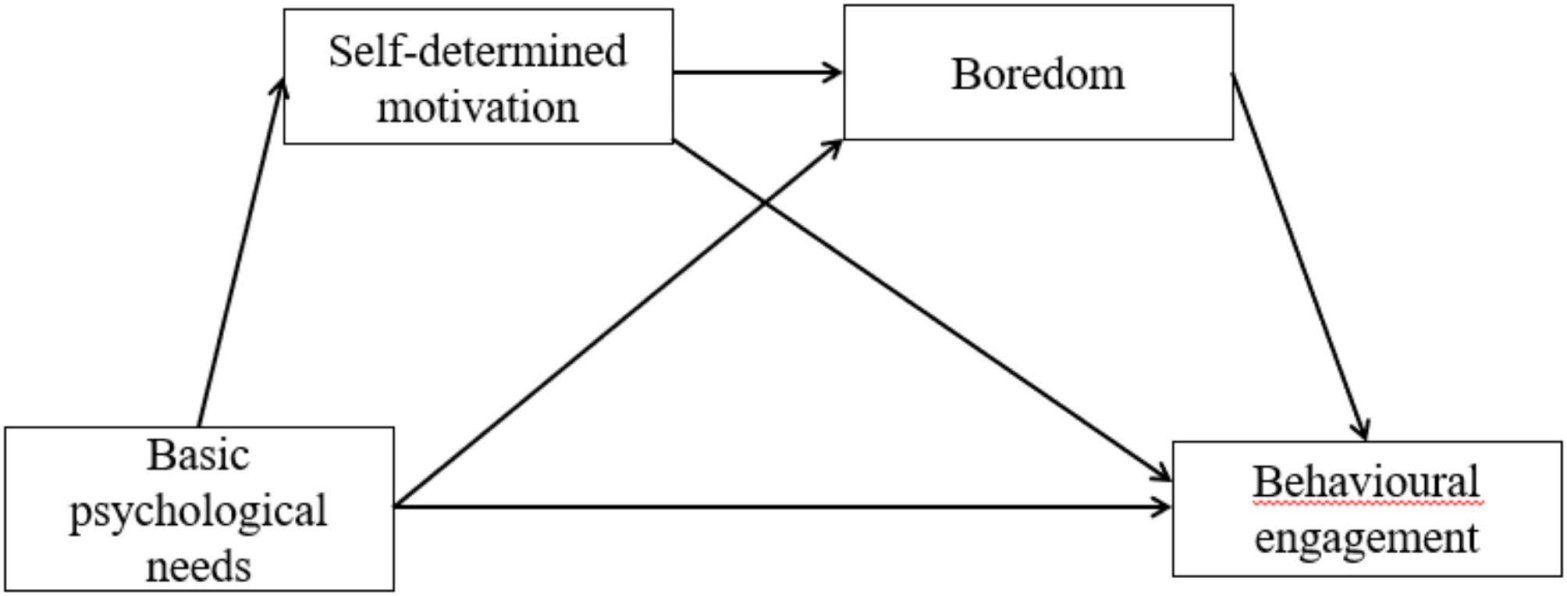
The Hypothesized Model

However, empirical studies in foreign language learning have revealed that the influence of basic psychological needs on behavioural engagement might be direct [37, 55] and indirect. Concerning the indirect link, for example, basic psychological needs may indirectly predict behavioural engagement through the mediating role of self-determined motivation (e.g [52, 80]). Basic psychological needs may also predict behavioural engagement through the mediating role of anxiety (e.g [33, 58]). However, the above influential mechanisms have not been comprehensively investigated. Among multiple EFL emotions, boredom, a ubiquitous emotion in EFL [70], is gradually attracting researchers’ attention and merits further investigation. Considering the similar effects of anxiety and boredom on EFL learning, such as behavioural engagement [28], and the principles of basic psychological needs theory and organismic integration theory, this study explores the complex relationship between basic psychological needs, self-determined motivation, boredom and behavioural engagement in the Chinese senior high school EFL context. The hypothetical model (see Fig. 1) and the corresponding research hypotheses are as follows:

1. Basic psychological needs directly affect students’ behavioural engagement in EFL learning.
2. Basic psychological needs indirectly affect students’ behavioural engagement in EFL learning, mediated through motivation.
3. Basic psychological needs indirectly affect students’ behavioural engagement in EFL learning, mediated through boredom.
4. Basic psychological needs indirectly affect students’ behavioural engagement in EFL learning, mediated through motivation and boredom.

## Research design

### Participants

The participants were 687 senior high school students from north-eastern parts of China. They comprised grade one (*N* = 283) and grade two students (*N* = 404). Concerning gender, the sample included 289 male and 398 female students. Their ages ranged from 16 to 18 years (*M* = 16.58, *SD* = 0.496). The students were all taking English as a compulsory course. They were from 21 classes in two cities in north-east China. They completed a unified examination before participating in this study. Due to the ethical concern of confidentiality, the schools did not offer data on the students’ EFL achievements. Therefore, the authors asked the EFL teachers to help in selecting the classes representing the higher, medium and lower levels of EFL achievement to ensure diversity in the participants’ EFL achievements.

### Instruments

The present study used a questionnaire to collect the data. The questionnaire consisted of an introduction, background information and the body. The introduction described the present study’s research aims, the request to complete the questionnaire, authorship and contact information. The background information section asked students to provide their gender, age, grade and school names. Four scales framed the body: basic psychological needs, motivation, boredom and behavioural engagement scales. These scales were five-point Likert scales, where responses 1 to 5 represented “totally disagree/extremely unlikely” to “totally agree/extremely likely”. After the questionnaire was developed, it was translated into Chinese for the convenience of fully understanding the content of each item. The authors translated all the original English items into Chinese and then performed a back translation [86]. After the translation, the authors invited experts in applied linguistics and two senior high school teachers to check the appropriateness of the translated scales.

The basic psychological needs scale was adapted from [54] and Leeming and Harris [87] and contained three dimensions: autonomy, competence and relatedness. Each dimension was composed of four items. In the present study, the model fit indices were satisfactory (χ^2^/*df* = 4.878, RMSEA = 0.075, SRMR = 0.020, CFI = 0.977, TLI = 0.968) for the 10-item measurement model including original three dimensions after items 6 (“I consider myself good at English”) and 12 (“I am working hard with my classmates”) were deleted to avoid collinearity according to the modification indices provided by Mplus. The factor loadings of the items were higher than 0.60. Cronbach’s alpha for the scale was 0.947, indicating good reliability. The high correlations between the three dimensions are consistent with the claim in SDT that fundamental needs are interdependent [43]. Therefore, the 10-item measurement model of the basic psychological needs scale was accepted.

Alamer’s [55] self-determination theory-L2 was used to measure EFL motivation. It comprised four dimensions: intrinsic motivation, identified motivation, introjected motivation and external motivation. Five items represented each dimension. In the measurement model with the original four factors, items 18, 24, 25, 26 and 31 were deleted for the cross-factor loadings or non-conformity with SDT. For instance, the item “because I want to get a prestigious job that requires English proficiency” was loaded on intrinsic motivation, thus conflicting with the principles of the organismic integration mini-theory in SDT. After combining two factors – intrinsic motivation and identified motivation – due to their high correlations, according to the model results provided by CFA, the model fit indices were acceptable (χ^2^/*df* = 6.950, RMSEA = 0.093, SRMR = 0.085, CFI = 0.919, TLI = 0.896) [88]. The factor loadings of items for the three dimensions were higher than 0.50. According to the organismic integration mini-theory in SDT, intrinsic and identified motivations are named autonomous motivation. Therefore, the new dimension was named “autonomous motivation”. Cronbach’s alphas for the three dimensions – autonomous motivation, introjected motivation and external motivation – were 0.933, 0.772 and 0.643, respectively, indicating good reliability. Thus, the three-factor motivation scale with 15 items was accepted.

Boredom was measured by adapting Bieleke et al.’s [89] boredom subscale in their achievement emotion questionnaire (short version). The boredom subscale included eight items measuring learning-related and class-related boredom. All eight items were revised to fit the present study. For instance, the original item “I get bored” was revised as “I get bored during English class”. In the measurement model, the model fit indices were satisfactory (χ^2^/*df* = 4.566, RMSEA = 0.072, SRMR = 0.005, CFI = 0.996, TLI = 0.989) for the 6-item unidimensional boredom scale after deleting items 37 (“I would rather put off this boring English course-related work till tomorrow”) and 38 (“studying for my courses bores me”) to avoid collinearity. The factor loadings of the six items were higher than 0.80. Cronbach’s alpha for the scale was 0.960, indicating good reliability.

The behavioural engagement scale was adapted from Reeve and Tseng’s [71] 5-item behavioural engagement subscale. All five items were revised to fit the present study. For instance, the original item “I listen carefully in class” was revised as “I listen carefully in English class”. In the measurement model, the model fit indices were satisfactory (χ^2^/*df* = 1.433, RMSEA = 0.025, SRMR = 0.005, CFI = 1.000, TLI = 0.998) for the 5-item unidimensional behavioural engagement scale. The factor loadings of the five items were higher than 0.75. Cronbach’s alpha for the scale was 0.921, indicating good reliability.

### Procedure

The authors uploaded the questionnaire to the online survey tool (www.wjx.cn) to collect the data. The QR code of the online questionnaire was sent to participants during their English classes, and they were asked to fill in the questionnaire after class. Before the questionnaire distribution, the authors contacted school leaders and class teachers. After obtaining their consent, the authors explained the purpose of this study to the students before they completed the questionnaire. Altogether, 687 valid responses were obtained after screening the data for time and invariant responses.

All the data collection procedures followed the ethical standards of quantitative research. Prior to the data collection, the students were fully informed of the purpose and details of the current study. They all completed the written consent form and voluntarily participated in this study. Specifically, all the participants completed the questionnaire voluntarily and were allowed to withdraw at any time of the research. This study also assured the anonymity of all participants, and their personal information and answers to the questionnaire were kept confidential and simply used for academic purposes without any negative impact on them.

For the data analysis, the normal distribution of the data was tested using SPSS 24.0 and then the measurement models were established. Several confirmatory factor analyses (CFAs) were conducted using Mplus 8.3 to test the measurement models. The benchmarks for the model fit indices were CMIN/DF ≤ 5, TLI ≥ 0.9, CFI ≥ 0.90, RMSEA ≤ 0.08 and SRMR ≤ 0.10 [90]. Regarding reliability, the threshold of 0.6 was adopted in testing Cronbach’s alpha using SPSS 24.0. In addition, a descriptive analysis was performed to describe the levels of variables under investigation. Pearson correlation analysis was also conducted before the mediation analysis was carried out using PROCESS Plug-in (MODEL6) in SPSS 24.0, following Hayes [91].

## Results

### Levels and correlations between variables

First, the normal distribution of the data was tested through the skewness and kurtosis of each variable. The results indicated the normal distribution of the data if the range of skewness and kurtosis varied between − 3∼ + 3 and − 10∼ + 10, respectively [92]. The second step was a descriptive analysis. For the level of motivation, with reference to SDT, which presents that the relative autonomy index (RAI) is an approach to evaluate the overall motivation quality with a single factor [43] and previous empirical studies (e.g [93])., the RAI was used to describe levels of self-determination of students’ EFL motivation. Then, following Hayes’ [91] advice, a preliminary correlation analysis was conducted to show the correlations between variables. The results are presented in Table 1.

**Table 1:** Means and correlation coefficients between variables

	M	SD	Skewness	Kurtosis	BPN	RAI	BO	BE
BPN	3.66	0.87	−0.59	0.43				
RAI	1.08	3.41	0.08	0.29	0.588^**^			
BO	2.50	1.05	0.56	−0.23	−0.545^**^	−0.583^**^		
BE	3.80	0.83	−0.67	0.81	0.817^**^	0.517^**^	−0.543^**^	

Note: **p < 0.01; BPN = basic psychological needs; RAI = relative autonomy index; BO = boredom; BE = behavioural engagement

Table 1 shows that all four variables under analysis were normally distributed, indicating that further analysis could be conducted. The descriptive analysis results showed that in English learning, senior high school students’ basic psychological needs were moderate to highly satisfied (*M* = 3.66, *SD* = 0.87). Their motivation to learn EFL was relatively autonomous as opposed to controlled (*M* = 1.08, *SD* = 3.41). In addition, the students experienced low levels of boredom (*M* = 2.50, *SD* = 1.05) and showed moderate to high behavioural engagement (*M* = 3.80, *SD* = 0.83).

The results of the correlation analysis revealed significant correlations between the variables. Students’ basic psychological needs were significantly and positively correlated with self-determined motivation and behavioural engagement (*r* = 0.588; *r* = 0.817, *p* < 0.01) and negatively correlated with boredom (*r* = − 0.545, *p* < 0.01). Students’ self-determined motivation was significantly and positively correlated with behavioural engagement (*r* = 0.517; *p* < 0.01) and negatively correlated with boredom (*r* = − 0.583, *p* < 0.01). Boredom and behavioural engagement were significantly and negatively correlated (*r* = 0.543, *p* < 0.01).

### Testing the hypothesized model

**Fig. 2:**
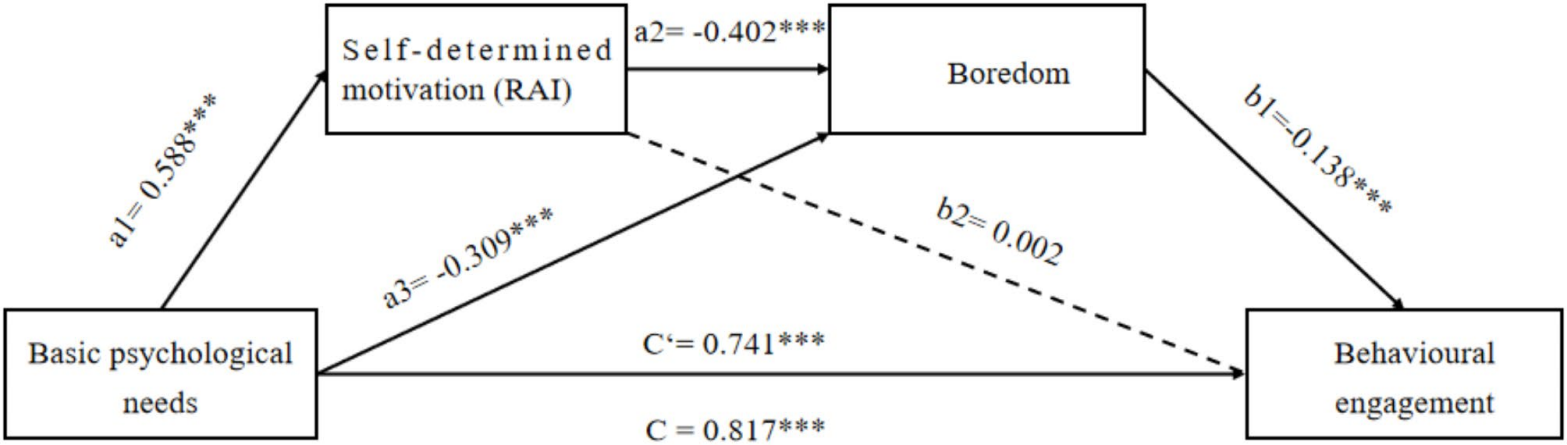
Path diagram of the mediation model

Next, a chain mediation analysis was carried out according to the hypothetical model, using basic psychological needs as the independent variable, behavioural engagement as the dependent variable and self-determined motivation and boredom as mediators. The results displayed that the influential path from self-determined motivation to behavioural engagement was non-significant (*p* > 0.05). The paths between other variables were significant (*p* < 0.001). The results are presented in Fig. 2.

The results of the mediation analysis confirmed that the chain mediation model was significant (R^2^ = 0.668, F(685) = 1376.547, *p* < 0.001). The direct and indirect effects and 5,000 random-sample bootstrapping confidence intervals (CIs) are summarized in Table 2. The results showed that the total effect of basic psychological needs on behavioural engagement was significant (B = 0.779, SE = 0.021, *p* < 0.001, 95%CI = 0.738, 0.820). The direct effect of basic psychological needs on behavioural engagement was also significant (B = 0.706, SE = 0.027, *p* < 0.001, 95%CI = 0.654, 0.759), indicating the partial mediating effects of self-determined motivation and boredom in the relationship between basic psychological needs and behavioural engagement.

Regarding the indirect effect of basic psychological needs on behavioural engagement, the results showed that the simple mediating role of self-determined motivation was non-significant (B = 0.001, SE = 0.016, 95%CI = − 0.029, 0.032), but the simple mediating role of boredom was significant (B = 0.041, SE = 0.015, 95%CI = 0.017, 0.074). Furthermore, the chain mediations of self-determined motivation and boredom were significant (B = 0.033, SE = 0.009, 95%CI = 0.015, 0.050). The results confirmed hypotheses 1, 3 and 4 and contradicted hypothesis 2.

**Table 2:** The direct and indirect paths between BPN and BE

Paths	estimate	SE	95% CIs	Relative effects
Total effect	0.779	0.021	[0.738, 0.820]	——
Direct effect	0.706	0.027	[0.654, 0.759]	90.6
Total indirect effect	0.073	0.022	[0.032, 0.119]	9.4
BPN→RAI→BE	0.001	0.016	[− 0.029, 0.032]	——
BPN→BO→BE	0.041	0.015	[0.017, 0.074]	5.3
BPN→RAI→BO→BE	0.031	0.009	[0.015, 0.050]	4.1

Note: BPN = basic psychological needs; RAI = relative autonomy index; BO = boredom; BE = behavioural engagement

## Discussion

The present study uncovered that students’ basic psychological needs directly predicted their behavioural engagement in the context of senior high school EFL learning. According to the claims of basic psychological needs theory in SDT, the satisfaction of basic psychological needs relates closely to people’s positive behaviours [43]. In other words, learners’ basic psychological needs are the direct antecedents of learning engagement [36]. The satisfaction of the needs for autonomy, competence and relatedness promotes active participation or effort in EFL learning [37, 55]. In the exam-oriented senior high school EFL context, satisfying autonomy, competence and relatedness is still vital to maintaining active behavioural engagement.

The present study also uncovered that the simple mediation of self-determined motivation in the relationship between basic psychological needs and behavioural engagement was non-significant, indicating that students’ basic psychological needs failed to influence their EFL behavioural engagement through self-determined motivation. This is inconsistent with the claim of SDT that the higher the self-determination, the more engagement there is in related activities. This may be because, in some cultural contexts, achievement in EFL learning is a determinant factor in personal development (e.g. higher social status) [82]. For the participants in this study, EFL learning was also crucial for achieving good scores on the college entrance examination, which would be closely connected with future jobs and self-development. Therefore, students with external motivation would actively engage in EFL learning [79] during senior high school, decreasing the degree of self-determination in EFL motivation. This result contradicts SDT as self-determined motivation was measured by the relative autonomy index. However, the high levels of both autonomous and controlled motivation featured Chinese senior high school EFL learners’ motivation. Consequently, self-determined motivation could not predict students’ EFL behavioural engagement, although basic psychological needs could significantly predict self-determined motivation [51, 52, 54, 55].

Another possible explanation for the above finding is that other mediating variables exist in the relationship between self-determined motivation and behavioural engagement, such as emotions. In many previous studies, self-determined motivation directly influenced learning engagement in EFL learning [9, 20, 79–81]. However, in some cases, there was no support for the argument that motivation is the direct source of learning engagement [36]. This is because some students are motivated but cannot devote much effort to EFL learning. For example, in the high-stakes Chinese senior high school EFL learning context, students must pursue other subjects to maintain their comprehensive academic performance. The finding that self-determined motivation could not predict EFL behavioural engagement contributes to the application of SDT in the language learning field by displaying its culture-specific and context-specific nature [43].

The present study also unveiled that the simple mediation of boredom in the relationship between basic psychological needs and behavioural engagement was significant, suggesting that students’ satisfaction of basic psychological needs will inhibit boredom, encouraging their EFL behavioural engagement. This agrees with the basic psychological needs theory: basic psychological needs satisfaction will induce positive feelings and attenuate negative affective status [43, 85]. In response, some empirical studies in SLA have also demonstrated that students’ basic psychological needs negatively predicted learner anxiety [58]. The anxiety would further negatively predict language learning engagement [33]. Given the same hindering roles of anxiety and boredom [27, 28], boredom could mediate the relationship between basic psychological needs and behavioural engagement.

Finally, in the current study, the chain mediating roles of self-determined motivation and boredom in the relationship between basic psychological needs and behavioural engagement were also significant, implying that the level of self-determination of EFL motivation improved with the satisfaction of basic psychological needs. Highly self-determined motivation further reduced boredom, motivating active behavioural engagement. This result demonstrates one of the reasons for the rejection of hypothesis 2 in this study. This could be explained by the SDT principles and empirical findings. Previous literature has shown a significant negative predictive role of boredom in behavioural engagement in EFL learning [27, 28, 33] and revealed the existence of close relationships between self-determined motivation and emotions [9] and between basic psychological needs and self-determined motivation [54, 94]. Basic psychological needs theory and organismic integration theory in SDT also support that the satisfaction of students’ basic psychological needs leads to highly self-determined motivation; this, in turn, decreases the levels of negative emotions that are among the predictive factors of learning behaviours. We could claim that students’ basic psychological needs are satisfied in EFL learning, and their motivation becomes more self-determined, which would further alleviate the experience of boredom and promote behavioural engagement.

## Conclusion

In conclusion, based on SDT and previous studies on basic psychological needs, self-determined motivation, boredom and behavioural engagement, the present study explored the relationship between these variables and testified to the direct predictive role of basic psychological needs in senior high school students’ EFL behavioural engagement and the simple mediating role of boredom as well as the chain mediating role of self-determined motivation and boredom in the relationship between basic psychological needs and behavioural engagement. For one thing, the present study’s contribution lies in revealing the significant role of boredom in the relationship between basic psychological needs, self-determined motivation and behavioural engagement, which extends the emotion research from the perspective of SDT. Another complex, influential mechanism between basic psychological needs and behavioural engagement was explored in the context of senior high school EFL to enrich the EFL engagement research. The results have practical implications for senior high school EFL teaching and learning:

First, students’ basic psychological needs should be satisfied. In this study, senior high school students’ basic psychological needs directly influenced their EFL behavioural engagement. In the Chinese senior high school EFL context, the satisfaction of the need for competence seems essential. However, the present study’s high correlations between autonomy, competence and relatedness suggest that students’ three basic psychological needs are equally important. Therefore, teachers should pay equal attention to satisfying all three basic psychological needs. Specifically, teachers can implement differentiated teaching according to students’ language learning abilities to fulfil the need for competence. They can also provide more choices of learning materials and sources to meet students’ need for autonomy while showing respect and empathy towards students to satiate the need for relatedness and thus promote EFL behavioural engagement.

Second, internalizing EFL motivation is another way to improve students’ behavioural engagement. Although self-determined motivation failed to influence EFL behavioural engagement directly, it was beneficial in reducing boredom, which was the direct antecedent of EFL behavioural engagement. The results of this study indicate that students who are motivated to learn EFL with a high degree of self-determination will likely cultivate positive learning psychology and increase their levels of effort, attention and participation. In an exam-oriented context, teachers can emphasize the significance of learning EFL for students’ personal development or create learning contexts that relate to students’ real-life experiences. Furthermore, positive feedback is needed for students in high-stake learning environments, which is valuable for promoting more self-determined motivation. It will help in avoiding boredom, which is closely related to a high level of behavioural engagement.

Last but not least, reducing boredom in EFL learning is worthwhile as it is an emotion with a direct relationship to EFL behavioural engagement. The exam-oriented EFL learning context will likely trigger boredom because of monotonous learning materials, a high-pressure learning atmosphere, etc. Teachers can target this characteristic and use emotional contagion to transmit enthusiasm for EFL learning to achieve low levels of boredom. This can be achieved by integrating multiple teaching methods and materials, making full use of online resources, planning activities that align with students’ abilities and providing positive feedback.

Despite the above implications, the present study had some limitiations. First, only behavioural engagement was examined as it is vital to the multidimensional learning engagement construct. Therefore, the relationships between basic psychological needs, self-determined motivation, boredom and other dimensions of engagement are worthy to be discussed in the future studies. Second, boredom was adopted as an example to discuss its mediating roles in the relationship between basic psychological needs, self-determined motivation and behavioural engagement. However, students experience diverse emotions in EFL learning, and they are worthy of further study [57]. Amotivation and integrated motivation were not explored in our study, but they are two valuable topics in the future studies which can examine the levels of these variables and their relationships with other psychological factors. Concerning the research methods, only quantitative self-measures were used to measure the four variables; therefore, the results of the present study need further triangulation by adopting other data collection methods, such as in-depth interviews. Another limitation is that this study was conducted in a senior high school EFL context in an area of China. Future research can recruit more students in other areas and consider the junior high school students as the partcipants to enhance the representativeness.

## Electronic supplementary material

Below is the link to the electronic supplementary material.

Supplementary Material 1

## Data Availability

The datasets and materials used and/or analysed during the current study are available from the corresponding author on reasonable request.

## Abbreviations

SLA: Second language acquisition
SDT: Self-determination theory
EFL: English as a foreign language
CFA: Confirmatory factor analysis
RAI: Relative autonomy index
CI: Confidence interval
