# Supplementary material for: Exploring the mediating roles of motivation and boredom in basic psychological needs and behavioural engagement in English learning: a self-determination theory perspective
Source: BMC Psychol. 2025 Feb 28;13:179. doi: 10.1186/s40359-025-02524-3 (PMC11871695; doi:10.1186/s40359-025-02524-3)
Supplement: Supplementary file 1 — Supplementary Material 1 [file 40359_2025_2524_MOESM1_ESM.pdf]

CFA Results for the Original and Modified Scales

basic psychological needs scale

Figure 1: original scale

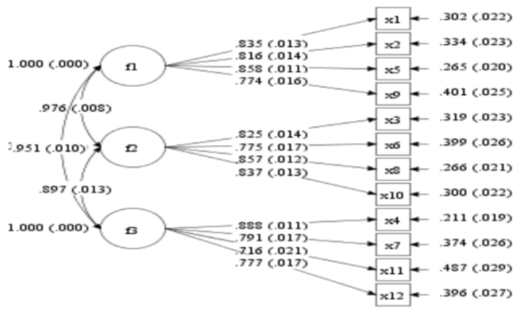

χ^2/df=8.562, RMSEA=0.105, SRMR=0.033,

CFI = 0.943, TLI = 0.927

Figure 2: modified scale 1 (single factor)

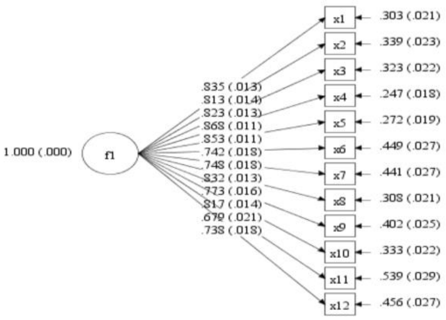

χ^2/df =10.463, RMSEA =0.117, SRMR= 0.039,

CFI = 0.925, TLI = 0.908

Figure 3: modified scale 2 (deletion of item 12)

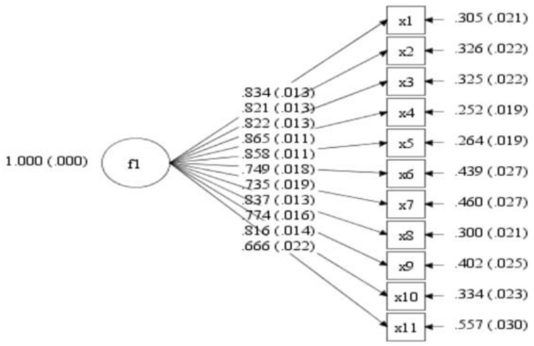

χ^2/df=9.030, RMSEA=0.108, SRMR=0.033,

CFI = 0.942, TLI = 0.928

Figure 4: modified scale 3 (deletion of item 6)

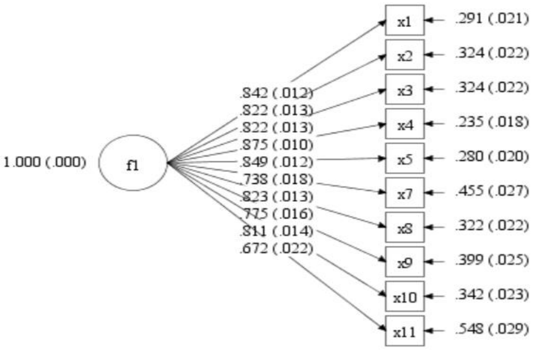

χ^2/df =7.097, RMSEA =0.094, SRMR= 0.028,

CFI = 0.961, TLI = 0.950

Figure 5: modified scale 4 (link between 7and 11)

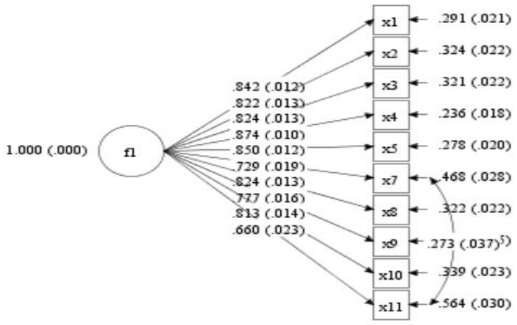

Figure 6: final scale (link between 9 and 10)

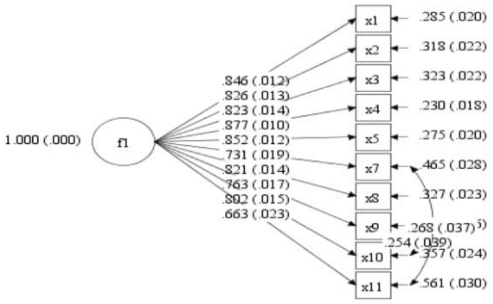

χ^2/df=5.871, RMSEA=0.084, SRMR=0.023, CFI = 0.970, TLI = 0.960

χ^2/df =4.878, RMSEA =0.075, SRMR= 0.020, CFI = 0.977, TLI = 0.968

Motivation scale

Figure 1: original scale

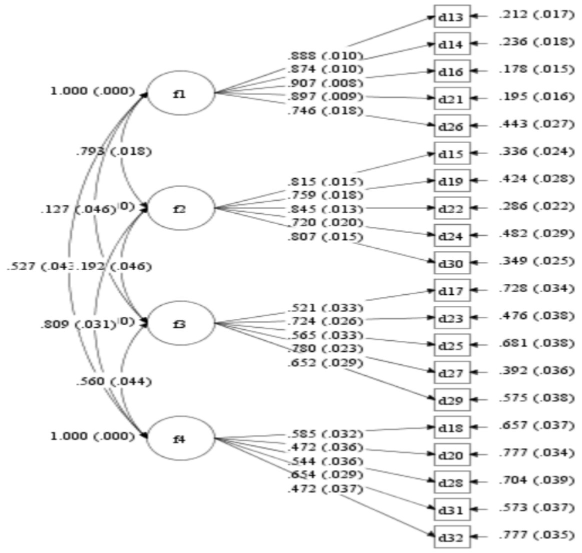

Figure 2: modified scale 1 (deletion of item 25)

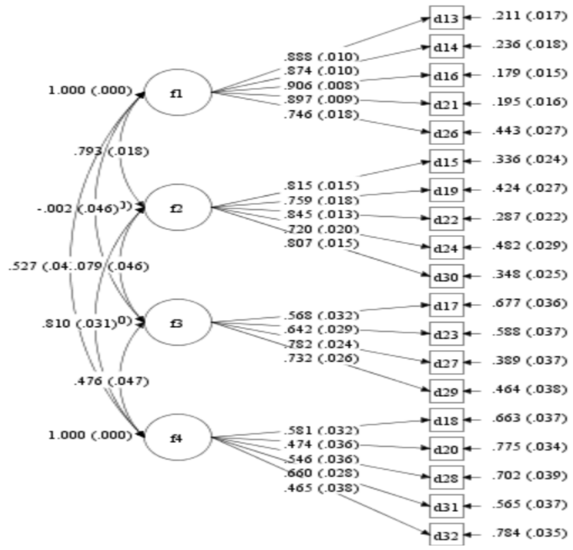

χ^2/df=9.650, RMSEA=0.112, SRMR=0.124, CFI = 0.834, TLI = 0.808

χ^2/df =8.881, RMSEA =0.107, SRMR= 0.107, CFI = 0.858, TLI = 0.834

Figure 3: modified scale 2 (deletion of item 26)

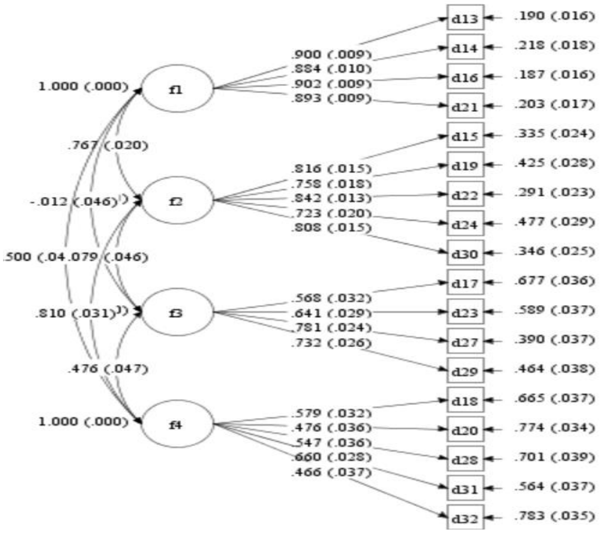

Figure 4: modified scale 3 (deletion of item 31)

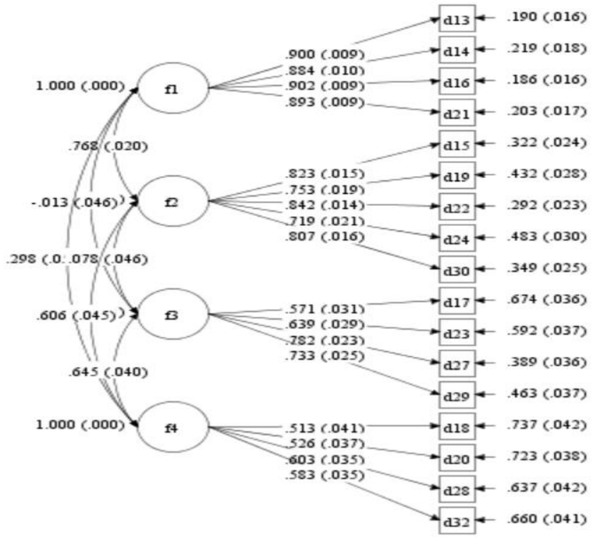

χ^2/df=8.413, RMSEA=0.104, SRMR=0.105, CFI = 0.871, TLI = 0.847

χ^2/df =7.621, RMSEA =0.098, SRMR= 0.103, CFI = 0.892, TLI = 0.870

Figure 5: modified scale 4 (deletion of item 18)

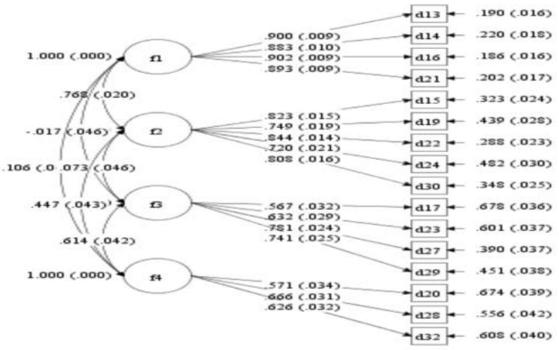

χ^2/df=5.924, RMSEA=0.085, SRMR=0.076,

CFI = 0.926, TLI = 0.910

Figure 6: modified scale 5 (deletion of item 24)

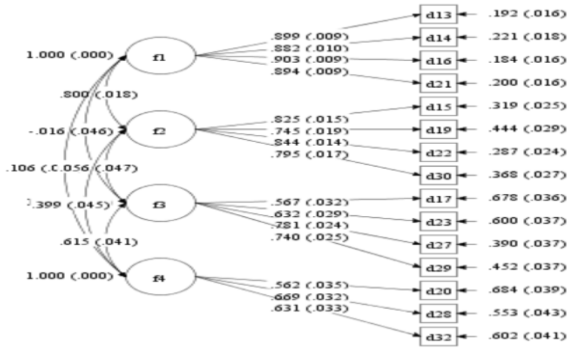

χ^2/df =5.134, RMSEA =0.078, SRMR= 0.070,

CFI = 0.942, TLI = 0.928

Figure 7: modified scale 6 (combination of f1 and f2)

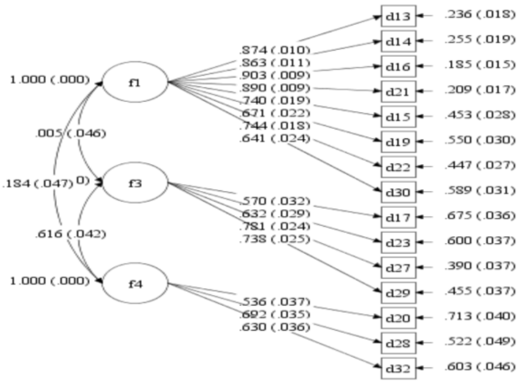

χ^2/df=10.656, RMSEA=0.119, SRMR=0.089,

CFI = 0.861, TLI = 0.831

Figure 8: modified scale 7 (link between 22 and 30)

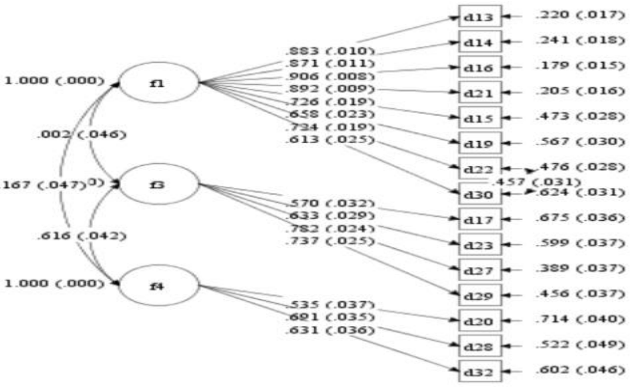

χ^2/df =9.061, RMSEA =0.108, SRMR= 0.088,

CFI = 0.884, TLI = 0.859

Figure 9: modified scale 8 (link between 13 and 14)

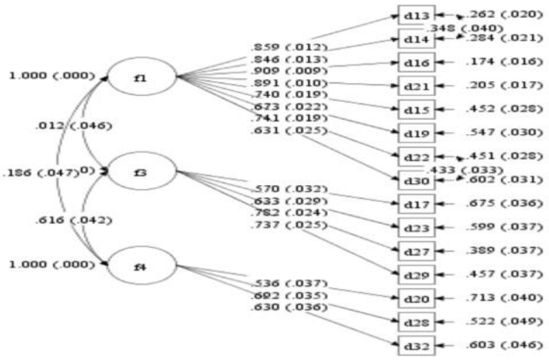

Figure 10: modified scale 9 (link between 15 with 30)

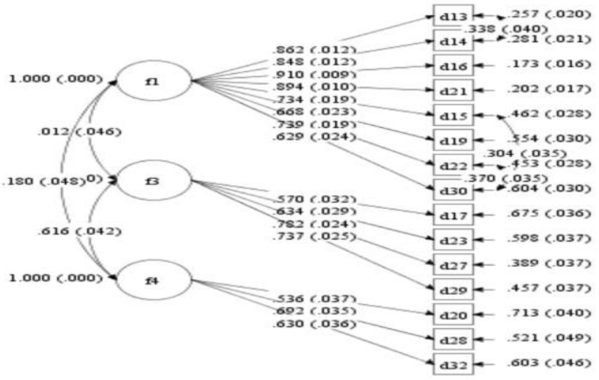

χ^2/df=8.472, RMSEA=0.104, SRMR=0.086,

CFI = 0.894, TLI = 0.869

χ^2/df =7.761, RMSEA =0.099, SRMR= 0.085,

CFI = 0.905, TLI = 0.882

Figure 11: final scale (link between 15 and 22)

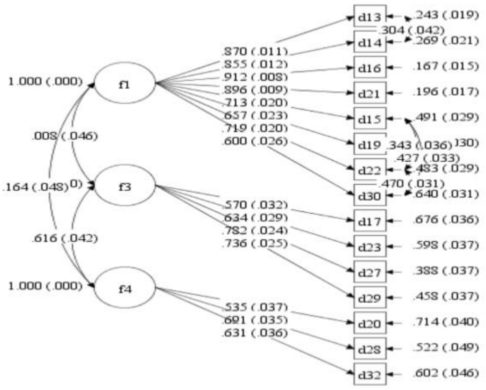

χ^2/df=6.950, RMSEA=0.093, SRMR=0.085,

CFI = 0.919, TLI = 0.896

Boredom scale

Figure 1: original scale

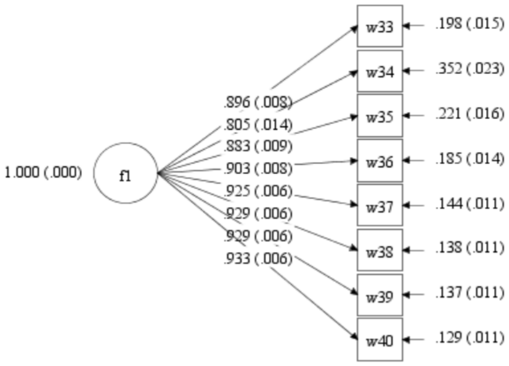

χ^2/df =18.506, RMSEA =0.160, SRMR= 0.023,

CFI = 0.952, TLI = 0.933

Figure 2: modified scale 1 (link between 39 and 40)

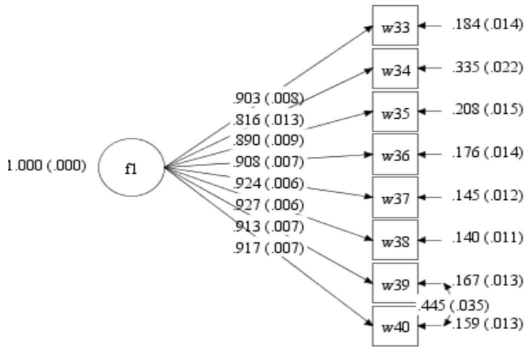

χ^2/df=13.621, RMSEA=0.136, SRMR=0.020,

CFI = 0.967, TLI = 0.952

Figure 3: modified scale 2 (link between 33 and 34)

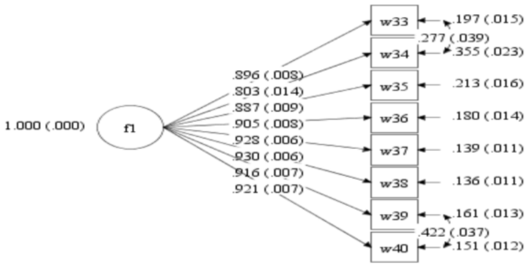

Figure 4: modified scale 3 (deletion of item 38)

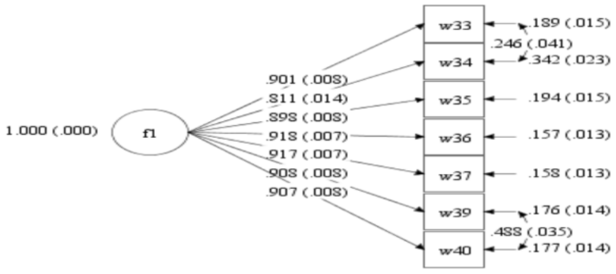

χ^2/df =11.884, RMSEA =0.126, SRMR= 0.018,

CFI = 0.973, TLI = 0.958

χ^2/df=10.736, RMSEA=0.119, SRMR=0.016,

CFI = 0.981, TLI = 0.966

Figure 5: modified scale 4 (link between 34 and 35)

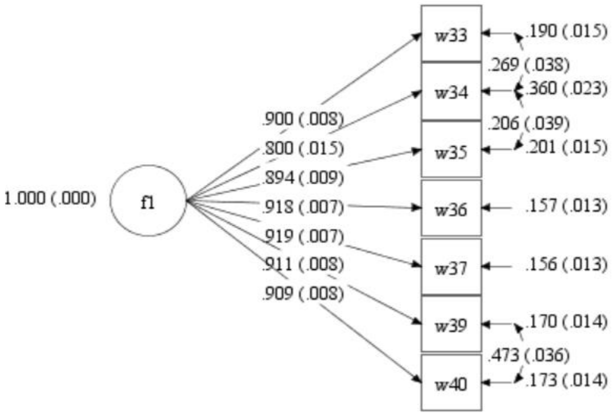

χ^2/df =9.351, RMSEA =0.110, SRMR= 0.013,

CFI = 0.985, TLI = 0.971

Figure 6: final scale (deletion of item 37)

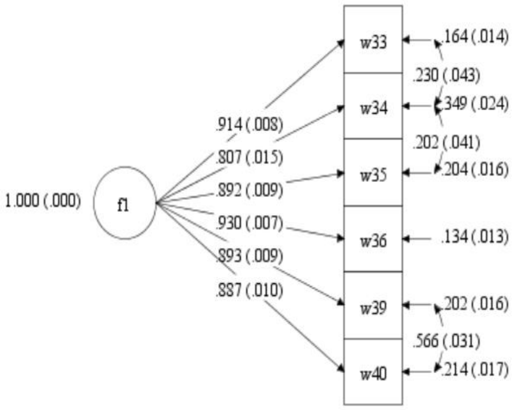

χ^2/df=4.566, RMSEA=0.072, SRMR=0.005,

CFI = 0.996, TLI = 0.989

behavioural engagement scale

Figure 1: original scale

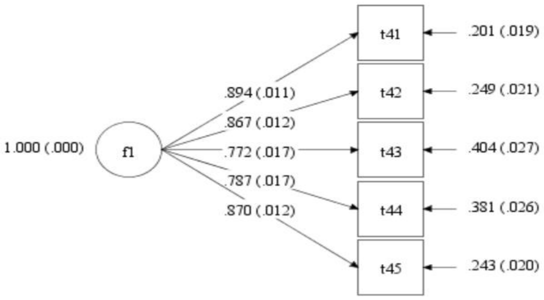

χ^2/df =31.504, RMSEA =0.211, SRMR= 0.036,

CFI = 0.942, TLI = 0.884

Figure 2: modified scale 1 (link between 41 and 42)

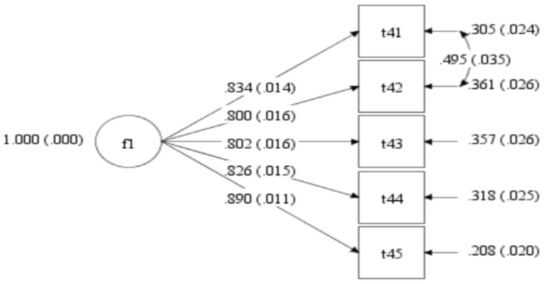

χ^2/df=10.817, RMSEA=0.120, SRMR=0.019,

CFI = 0.985, TLI = 0.963

Figure 3: final scale

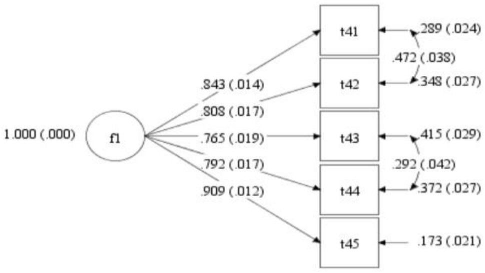

χ^2/df =1.433, RMSEA =0.025, SRMR= 0.005,

CFI = 1.000, TLI = 0.998
